# FilGAP regulates tumor growth in Glioma through the regulation of mTORC1 and mTORC2

**DOI:** 10.1038/s41598-023-47892-1

**Published:** 2023-12-08

**Authors:** Koji Tsutsumi, Ayumi Nohara, Taiki Tanaka, Moe Murano, Yurina Miyagaki, Yasutaka Ohta

**Affiliations:** Division of Cell Biology, Department of Biosciences, School of Science, Kitasato University, 1-15-1 Kitasato, Sagamihara, Minami-Ku, Kanagawa 252-0373 Japan

**Keywords:** TOR signalling, Mechanisms of disease

## Abstract

The mechanistic target of rapamycin (mTOR) is a serine/threonine protein kinase that forms the two different protein complexes, known as mTORC1 and mTORC2. mTOR signaling is activated in a variety of tumors, including glioma that is one of the malignant brain tumors. FilGAP (ARHGAP24) is a negative regulator of Rac, a member of Rho family small GTPases. In this study, we found that FilGAP interacts with mTORC1/2 and is involved in tumor formation in glioma. FilGAP interacted with mTORC1 via Raptor and with mTORC2 via Rictor and Sin1. Depletion of FilGAP in KINGS-1 glioma cells decreased phosphorylation of S6K and AKT. Furthermore, overexpression of FilGAP increased phosphorylation of S6K and AKT, suggesting that FilGAP activates mTORC1/2. U-87MG, glioblastoma cells, showed higher mTOR activity than KINGS-1, and phosphorylation of S6K and AKT was not affected by suppression of FilGAP expression. However, in the presence of PI3K inhibitors, phosphorylation of S6K and AKT was also decreased in U-87MG by depletion of FilGAP, suggesting that FilGAP may also regulate mTORC2 in U-87MG. Finally, we showed that depletion of FilGAP in KINGS-1 and U-87MG cells significantly reduced spheroid growth. These results suggest that FilGAP may contribute to tumor growth in glioma by regulating mTORC1/2 activities.

## Introduction

The mechanistic target of rapamycin (mTOR) is a serine/threonine protein kinase that is highly conserved from yeast to mammals belonging to the PI3K-related protein kinases family. mTOR forms functionally distinct protein complexes, mTORC1 and mTORC2. mTORC1 has Raptor, and mTORC2 has Rictor and Sin1 as specific subunits respectively^1–4^. mTORC1 is activated by amino acids, and regulates protein synthesis and autophagy by phosphorylating downstream substrates, such as S6 kinase and 4EBP1^5–8^. mTORC2 is primarily activated by growth factors such as insulin, and regulates cell proliferation, cell survival, and cell migration by phosphorylating AKT, PKC and other substrates^9–14^. While the detailed mechanism of mTORC1 activation has been clarified, the molecular mechanism of mTORC2 activation remains unclear. Since new regulators of mTORC1 and mTORC2 activity have been reported even recently, the regulatory pathway of mTOR is considered to be very complex^14–20^. mTOR signaling is activated in a variety of tumors^4,21,22^. In particular, in glioma, one of the malignant brain tumors, activation of the mTOR pathway promotes cell proliferation and invasion, and contributes to patient poor prognosis^23–26^.

Rho small GTPases cycle between active GTP-bound and inactive GDP-bound state, and act as key regulators of actin cytoskeleton. Guanine nucleotide exchange factors (GEFs) activate Rho GTPase by catalyzing the exchange of GDP for GTP. While GTPase-activating proteins (GAPs) stimulate the intrinsic GTPase activity and inactivate them^27^. FilGAP (also known as ARHGAP24) is a Filamin A-binding protein, and Rac-specific GAP^28–31^. FilGAP suppresses leading edge protrusion and promotes cell retraction by inhibiting Rac and also regulates epithelial cell–cell adhesion and tissue morphogenesis^32–35^. In addition, it has been suggested that the expression level of FilGAP is associated with malignant transformation of cancer and is also involved in invasive metastasis and tumorigenesis^36–41^. However, the contribution of FilGAP to cancer development is thought to be different among cancer types, and more detailed mechanisms need to be elucidated.

In this study, we found that FilGAP interacts with mTORC1 and mTORC2. We showed that Raptor, Rictor and mTOR were coprecipitated with FilGAP. Depletion of FilGAP expression in KINGS-1 gliomas had an inhibitory effect on S6K and AKT phosphorylation, while overexpression of FilGAP had a promotive effect, suggesting that FilGAP activates mTORC1 and mTORC2. A short FilGAP isoform lacking partial PH domain was highly expressed in gliomas, and this short isoform also contributed to mTORC1 and mTORC2 activation. Depletion of FilGAP did not affect mTOR activity in U-87MG, a highly malignant glioblastoma, but significantly decreased AKT phosphorylation in the presence of a PI3K inhibitor. Finally, we showed that depletion of FilGAP inhibited spheroid formations in KINGS-1 and U-87MG as well as mTOR inhibition. These results suggest that FilGAP interacts with mTORC1 and mTORC2 and may positively regulate their activity to promote tumorigenesis in gliomas.

## Results

### FilGAP interacts with mTORC1 and mTORC2

**Figure 1:**
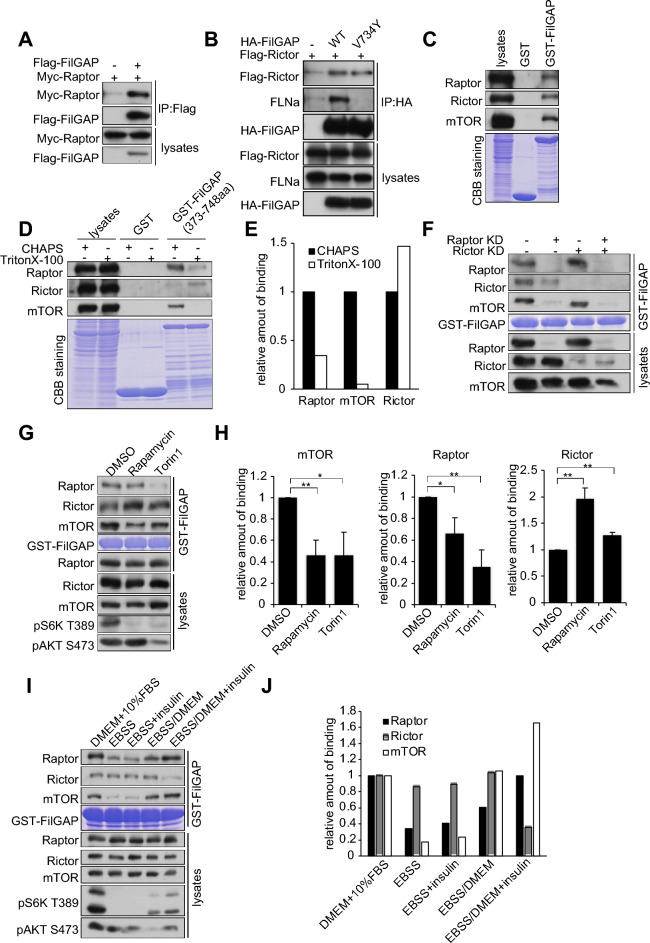
FilGAP interacts with mTORC1/2. (**A**) HEK293T cells were transfected with Flag-FilGAP and Myc-Raptor. After precipitation with Flag antibody, coprecipitated Raptor was detected with anti-Raptor antibody. (**B**) HEK293T cells were transfected with Flag-FilGAP (WT or V734Y) and Myc-Rictor. After precipitation with HA antibody, coprecipitated Rictor was detected with anti-Rictor antibody. (**C)** The purified recombinant GST-FilGAP (373-748aa) or GST alone were coupled to glutathione sepharose, and incubated with HEK293T cells lysed in buffer containing CHAPS. The washed precipitates were immunoblotted for the presence of Raptor, Rictor and mTOR. (**D)** GST-FilGAP (373-748aa) coupled to glutathione sepharose were incubated with HEK293T cells lysed in buffers containing CHAPS or Triton X-100. The washed precipitates were immunoblotted for the presence of Raptor, Rictor and mTOR. (**E**) Relative amount of coprecipitated Raptor, mTOR and Rictor in (**E**) was shown. (**F**) siRNAs against Raptor or Rictor were transfected HEK293T cells. 72 h later, cells were lysed in buffer containing CHAPS. Cell lysates were incubated with GST-FilGAP coupled to glutathione sepharose, and the washed precipitates were immunoblotted for the presence of Raptor, Rictor and mTOR. (**G**) HEK293T cells were treated with 100 nM Rapamycin or 500 nM Torin-1 for 1 h. Cell lysates were subjected to GST pull down assay with GST-FilGAP 373-748aa. (**H**) The amount of Raptor, Rictor, and mTOR precipitated with FilGAP in (**G**) was calculated and presented as the mean ± SE. *P < 0.05 **P < 0.01 (Student’s t-test). (**I**) HEK293T cells were cultured in EBSS without amino acids and serum for 2 h, and then incubated in DMEM medium or DMEM medium with insulin for 30 min. Cell lysates were subjected to GST pull down assay with GST-FilGAP 373-748aa. (**J**) The amount of Raptor, Rictor, and mTOR precipitated with FilGAP in (**I**) was calculated.

Recently, it has been reported that mTORC2 interacts with an actin filament crosslinking protein, Filamin A in glioblastoma cells^18,26^. Furthermore, mTOR has been reported to interact with various Rho family GTPases and their regulators^14,15,42,43^. Thus, we considered the possibility that FilGAP interacts with mTORC2 through Filamin A. To examine whether FilGAP interacts with mTOR complex, we transfected Raptor or Rictor with or without FilGAP in HEK293T cells and performed coimmunoprecipitation assay (Fig. 1A,B). Both Raptor and Rictor were coprecipitated with FilGAP, suggesting that FilGAP interacts with Raptor and Rictor. Rictor was also coprecipitated with FilGAP V734Y, Filamin A-binding deficient mutant, suggesting that this interaction is independent of Filamin A. Further, we investigated whether mTOR components in HEK293T cell lysates were pulled down with GST-FilGAP (373-748aa) (Fig. 1C). mTOR, Raptor and Rictor were co-precipitated with GST-FilGAP. This result suggests, that FilGAP may interact with both mTORC1 and mTORC2. The mTOR complexes are disrupted in the buffer with non-ionic detergent such as TritonX-100, but can be maintained in the buffer containing amphoteric detergent CHAPS^3^. Therefore, we prepared cell lysates with TritonX-100 or CHAPS and performed GST-pulldown assay (Fig. 1D). In the presence of TritonX-100, the coprecipitation with GST-FilGAP was decreased for Raptor and mTOR, but slightly increased for Rictor. We further examined the interaction of mTOR complex with FilGAP in Raptor- or Rictor-depleted cells (Fig. 1E). Knockdown of Raptor, but not Rictor, reduced the binding of mTOR to FilGAP, suggesting that most of the mTOR coprecipitated with FilGAP is mediated by Raptor. We examined whether binding to FilGAP is altered when the kinase activity of the mTOR is suppressed (Fig. 1F,G). Treatment of the cells with Rapamycin, mTORC1 specific inhibitor, and Torin1, mTOR inhibitor significantly decreased the amount of Raptor and mTOR coprecipitated with FilGAP. On the other hand, Rictor coprecipitated with FilGAP was rather increased by the inhibitor treatments. Similar data were observed when mTORC1 was activated by amino acids and insulin stimulation. (Fig. 1I,J and Fig. S1). These data suggest that the interaction of mTORC1 and FilGAP is dependent on mTORC1 activity.

### Identification of domains mediating FilGAP-mTORC1/2 interaction

**Figure 2:**
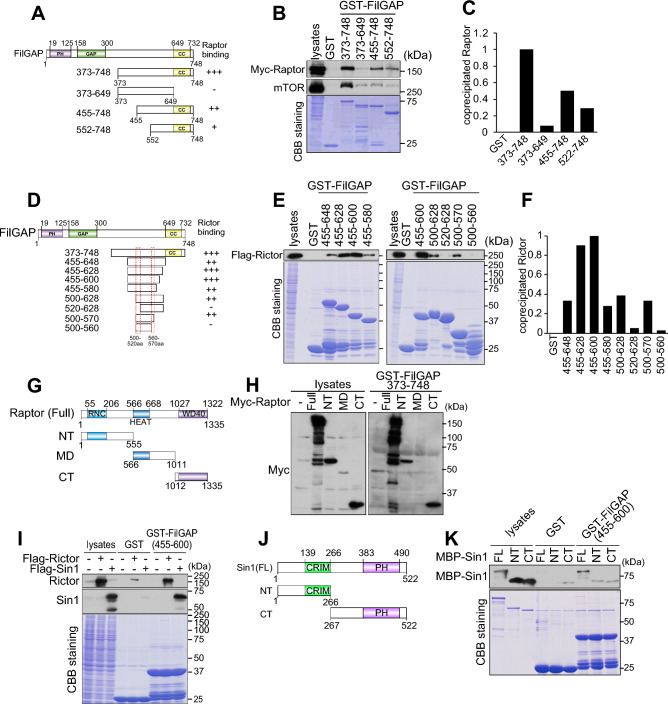
Analysis of FilGAP and mTORC1/2 association region. (**A**) Schematic diagram of FilGAP deletion mutants used to examine the interaction with Raptor. (**B**) Cell lysates prepared from Myc-Raptor transfected HEK293T cells were incubated with GST-FilGAP bound beads. Coprecipitated Myc-Raptor and mTOR were detected by immunoblotting. (**C**) Coprecipitated Raptor in (**B**) was quantified. (**D**) Schematic diagram of FilGAP deletion mutants used to examine the interaction with Rictor. The red dotted line is the estimated Rictor binding region. (**E**) Cell lysates prepared from Flag-Rcitor transfected HEK293T cells were incubated with GST-FilGAP bound beads. Coprecipitated Flag-Rictor was detected by immunoblotting. (**G**) Schematic diagram of Raptor deletion mutants. (**H**) Raptor NT and CT was coprecipitated with GST-FilGAP 373-748aa. (**I**) Flag-Rictor or Flag-Sin1 was transfected to HEK293T cells and cell lysates were subjected to pull down assay using GST-FilGAP 455-600aa. (**J**) Schematic diagram of Sin1 deletion mutants. (**K**) Purified MBP-Sin1 Full and NT was pulled down by GST-FilGAP 455-600aa.

To clarify the manner of the interaction between FilGAP and mTOR complexes, we examined detailed interaction between FilGAP and mTOR components. We generated a series of FilGAP-deletion mutants and examined their binding regions to Raptor or Rictor (Fig. 2A-F, Fig. S2A, B). C-terminus deletion resulted in a substantial decrease in interaction with Raptor, suggesting that the C-terminal region of FilGAP is important for binding to Raptor (Fig. 2A-C, Fig. S2A, B). On the other hand, it is suggested that FilGAP 500-520aa and 560-570aa are important for binding to Rictor (Fig. 2D-F). We generated deletion mutants of Raptor and examined their binding to GST-FilGAP and found that it binds to NT and CT but not to MD (Fig. 2G,H, Fig. S2C). Sin1, a specific component of mTORC2, also coprecipitated with FilGAP and the N-terminus of Sin1 was important for this binding. (Fig. 2I-K, Fig. S2D). Sin1 and FilGAP also co-precipitated in binding experiments with purified proteins, suggesting that this binding is direct (Fig. 2K).

### FilGAP activates mTORC1/2

**Figure 3:**
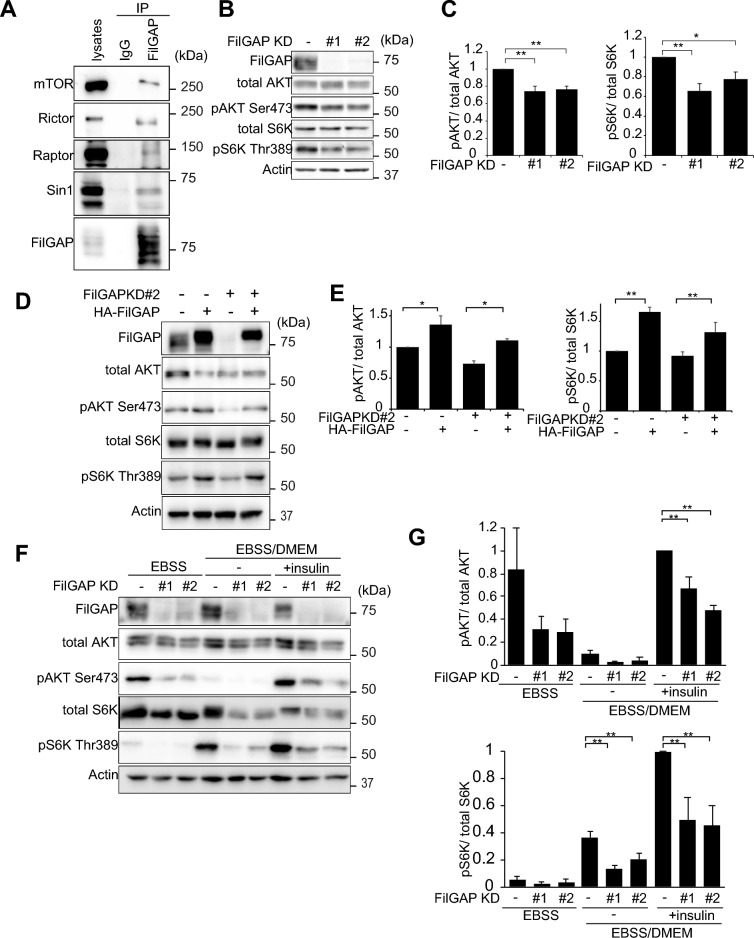
FilGAP activates mTORC1/2. (**A**) Endogenous FilGAP was immunoprecipitated from human astrocytoma KINGS-1 cell lysates and analyzed by immunoblotting. Normal rabbit IgG (IgG) was used as a control for immunoprecipitation. (**B**) Human astrocytoma KINGS-1 cells were transfected with siRNAs against FilGAP. Immunoblots for P-S6K and P-AKT represent the activities of mTORC1 and mTORC2 respectively. Actin was used as the loading control. (**C**) P-S6K/Total S6K and P-AKT/Total AKT ratio in (**B**) were calculated (n = 6). *P < 0.05, **P < 0.01 (ANOVA Tukey HDS Test). (**D**) control or FilGAP-depleted KINGS-1 cells were transfected with HA-FilGAP resistant to FilGAP siRNA KD#2 (FilGAP rKD#2). Actin was used as the loading control. (**E**) P-S6K/Total S6K and P-AKT/Total AKT ratio in (**D**) were calculated (n = 5). *P < 0.05, **P < 0.01 (Student’s t-test). (**F**) Human lung cancer A549 cells were transfected with siRNAs against FilGAP. 48 h after transfection, cells were cultured in EBSS without amino acids and serum for 2 h, and then incubated in DMEM medium or DMEM medium with insulin for 30 min. Immunoblots for P-S6K and P-AKT represent the activities of mTORC1 and mTORC2 respectively. Actin was used as the loading control. (**G**) P-S6K/Total S6K and P-AKT/Total AKT ratio in (**F**) were calculated (n = 3). *P < 0.05, **P < 0.01 (Student’s t-test).

To study the role of interaction between mTORC1/2 and FilGAP, we used KINGS-1 human astrocytoma cell line, which expresses high level of FilGAP^36^. To confirm whether FilGAP associates with mTORC1/2 in glioma cells, endogenous FilGAP was immunoprecipitated from KINGS-1 cells and confirmed the coprecipitation of mTORC1/2 components (Fig. 3A). Small interference RNAs (siRNAs) targeting FilGAP were transfected to KINGS-1 cells. Depletion of FilGAP in KINGS-1 cells significantly reduced phosphorylation of S6K and AKT, which are substrates of mTORC1 and mTORC2, respectively (Fig. 3B,C). Furthermore, transfection of HA-tagged FilGAP in KINGS-1 cells increased phosphorylation of S6K and AKT, and rescued the decrease in their phosphorylation caused by depletion of FilGAP (Fig. 3D,E). We next examined whether FilGAP is also involved in the activation of mTOR by amino acids and insulin. For this experiment, we used A549 lung cancer cell line, which is highly responsive to insulin. When the cells were cultured in amino acids-free EBSS (Earle’s Balanced Salt solution), phosphorylation of S6K was decreased (Fig. 3F). When the cells were returned to DMEM, a normal medium containing amino acids, phosphorylation of S6K was increased and phosphorylation of AKT was decreased. This may be due to the inhibition of mTORC2 by mTORC1 activation^44^. Furthermore, when the cells were treated with insulin, phosphorylation of both S6K and AKT was greatly increased. Phosphorylation of S6K and AKT induced by amino acids and insulin was significantly reduced by suppression of FilGAP expression (Fig. 3F,G). These results suggest that FilGAP activates mTORC1/2.

### PH domain of FilGAP is important for regulation of mTORC2 activity

**Figure 4:**
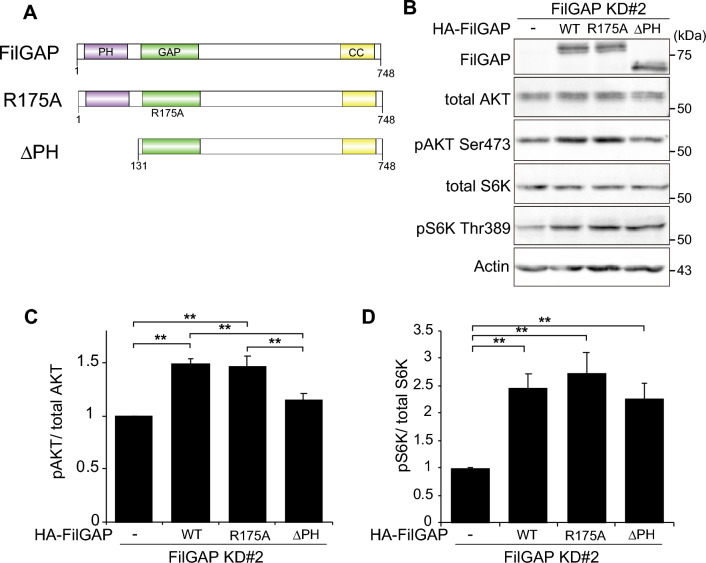
PH domain of FilGAP is important for regulation of mTORC2 activity. (**A**) Schematic diagram of FilGAP constructs. (**B**) KINGS-1 cells were transfected with HA-FilGAP rKD#2 constructs (WT, R175A or ΔPH) after depletion of FilGAP with siRNA. (**C**, **D**) P-AKT/Total AKT and P-S6K/Total S6K ratio in (**B**) were calculated (n = 6). *P < 0.05, **P < 0.01 (Student’s t-test).

FilGAP contains pleckstrin homology (PH), Rho GAP, and coiled-coil (CC) domains^28^. FilGAP R175A has mutation at GAP domain and lacks GAP activity. PH domain of FilGAP is required for Phosphatidylinositol 3-phosphate (PIP3)-dependent membrane localization^45^. To study whether GAP activity and PH domain of FilGAP is required for the regulation of mTORC1/2 activities, HA-FilGAP constructs (WT, R175A or ΔPH; Fig. 4A) resistant to FilGAP siRNA were transfected to KINGS-1 cells after depletion of endogenous FilGAP with siRNA (Fig. 4B). Although FilGAP wild-type (WT) and R175A significantly increased AKT phosphorylation, FilGAP ΔPH did not increase AKT phosphorylation as much as WT and R175A (Fig. 4C). On the other hand, all FilGAP constructs increased S6K phosphorylation to the same extent (Fig. 4D). These results suggest that PH domain of FilGAP is important for the regulation of mTORC2 activity.

### FilGAP partial PH-deleted transcript isoform activates mTORC1/2

**Figure 5:**
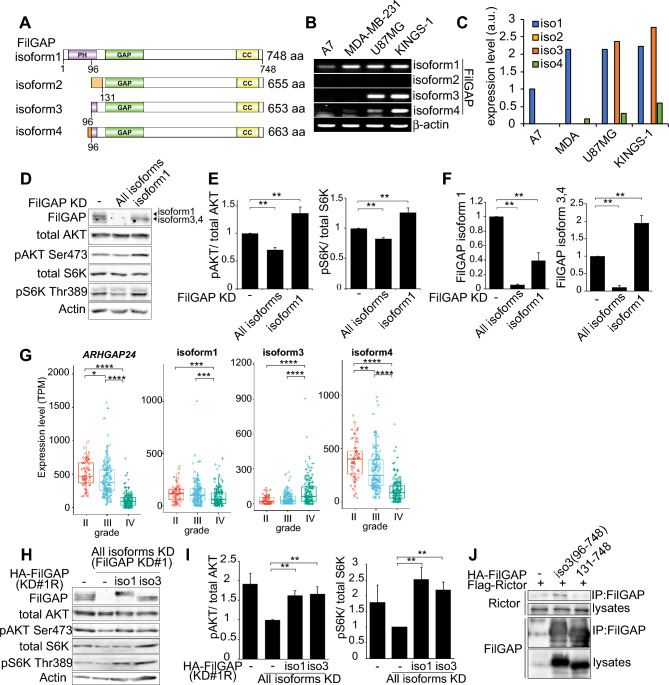
FilGAP partial PH-deleted isoform activates mTORC1/2. (**A**) Schematic diagram of FilGAP isoforms expressed in KINGS-1 cells. (**B**) RT-PCR analysis of expression of FilGAP isoforms in A7 melanoma, MDA-MB-231 breast cancer, U87MG glioma and KINGS-1 glioma cell line. β-actin was used as a loading control. (**C**) Quantification of band intensity in (**B**). (**D**) KINGS-1 cells were transfected with siRNA targeted against all FilGAP isoforms (FilGAP KD#1) or only isoform 1. (**E**) P-S6K/Total S6K and P-AKT/Total AKT ratio in (**D**) were calculated (n = 3). (**F**) FilGAP isoform expressions in (**D**) were calculated. (**G**) Box and jitter plots show expression level of overall *ARHGAP24*, isoform1, isoform3, and isoform4 in grade II (Astrocytoma), III (Anaplastic Astrocytoma), and IV (Glioblastoma multiforme). The data was obtained from TCGA database. Significance was determined using a Kruskal–Wallis test followed by a Dunn’s multiple comparisons test. (**H**) KINGS-1 cells were transfected with HA-FilGAP rKD#1 constructs (WT, 96-748aa) after depletion of FilGAP with siRNA. (**I**) P-S6K/Total S6K and P-AKT/Total AKT ratio in (**H**) were calculated (n = 8). (**J**) HEK293T cells were transfected with HA-FilGAP (isoform 3: 96-748aa or ΔPH:131-748 aa) and Flag-Rictor. 24 h after transfection, cells were lysed and after immunoprecipitation with anti-FilGAP antibody. *P < 0.05, **P < 0.01 , ***P < 0.001, ****P < 0.0001(Student’s t-test, **E**, **F**, **I**).

There are multiple FilGAP transcript isoforms, some of which are partially or completely lacking PH domain (Fig. 5A)^46,47^. The expression of FilGAP isoforms was examined by RT-PCR analysis and isoform 3 and 4 were highly expressed in gliomas (U-87MG and KINGS-1) compared to melanoma (A7) and breast cancer cells (MDA-MB-231) (Fig. 5B,C). To study whether FilGAP isoform 3 and 4 also regulate mTOR activity, siRNA targeting specific FilGAP isoform 1 was transfected to KINGS-1 cells. Knockdown of FilGAP isoform 1–4 (all isoforms) and only FilGAP isoform 1 were confirmed by immunoblotting (Fig. 5D). Depletion of all FilGAP isoforms by siRNA KD#1 decreased phosphorylation of S6K and AKT, while depletion of only FilGAP isoform 1 significantly increased their phosphorylation. (Fig. 5D, E). In addition, depletion of only FilGAP isoform 1 tended to increase the expression levels of isoform 3 and 4 (Fig. 5F). We investigated whether the expression level of FilGAP isoform changes with malignant transformation of gliomas using TSVdb, a web-based tool that can analyze the expression level of splicing variants from TCGA RNAseq data^48^. Overall FilGAP (*ARHGAP24*) expression was significantly decreased in glioblastoma (IV) compared to lower grade glioma (II and III) (Fig. 5G). This is consistent with the results of immunostaining of patient brains^36^. On the other hand, while isoform 1 and isoform 4 were decreased, isoform 3 was increased in glioblastoma compared to lower grade glioma. To further investigate the effect of FilGAP isoform 3 on the activities of mTORC1/2, HA-FilGAP constructs (96-748aa; isoform) resistant to FilGAP siRNA was transfected to KINGS-1 cells after depletion of FilGAP with siRNA. FilGAP isoform 3 increased mTORC1/2 activities to the same extent as isoform 1 (Fig. 5H,I). Interestingly, FilGAP isoform 3 showed increased coimmunoprecipitation with Rictor rather than ΔPH (131–748 aa) (Fig. 5J). These results suggest that FilGAP isoform 3 may have a higher or equal ability to activate mTORC1/2 compared to isoform 1. Give that ΔPH (131-748aa) cannot increased mTORC2 activity, 96–130 aa of FilGAP may be important to activate mTORC2.

### FilGAP regulates mTORC2 activity in U-87MG glioblastoma cell in the presence of PI3K inhibitor

**Figure 6:**
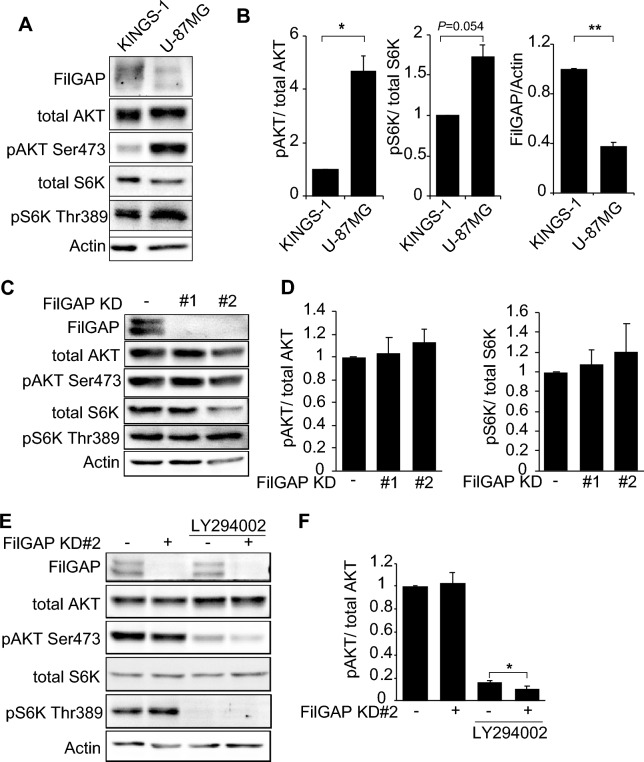
FilGAP is involved in PI3K-independent mTORC2 activation in glioblastoma cells. (**A**) Immunoblots for FilGAP expression and mTORC1/2 activities in astrocytoma KINGS-1 and glioblastoma U-87MG cells. (**B**) P-S6K/Total S6K, P-AKT/Total AKT ratio and amount of FilGAP expression in (A) were calculated (n = 3). (**C**) U-87MG cells were transfected with siRNAs against FilGAP. (**D**) P-S6K/Total S6K and P-AKT/Total AKT ratio in (**C**) were calculated (n = 3). (**E**) Control or FilGAP-depleted U-87MG cells were treated with 10 μM LY294002 for 1 h. (**F**) P-AKT/Total AKT ratio in (**E**) were calculated (n = 5). *P < 0.05, **P < 0.01 (Student’s t-test).

Gliomas are classified from grade II to grade IV according to histological and genetic diagnosis^49^. Grade IV glioblastoma is the most aggressive and has a very poor prognosis with a 2-year survival rate of less than 30%. To investigate the relationship between the mTORC1/2 regulation by FilGAP and grade of glioma malignancy, we compared mTORC1/2 activities and the expression levels of FilGAP in KINGS-1 and U-87MG, grade III astrocytoma and grade IV glioblastoma, respectively. Phosphorylation of S6K and AKT were higher in U-87MG than in KINGS-1, suggesting that mTORC1 and mTORC2 were more active in U-87MG. (Fig. 6A). The expression levels of FilGAP was higher in KINGS-1 cells than in U-87MG (Fig. 6B). To study whether FilGAP regulates mTORC1/2 activities in U-87MG cells as observed in KINGS-1, siRNAs targeting FilGAP was transfected to U-87MG and examined phosphorylation of AKT and S6K. Depletion of FilGAP in U-87MG cells did not affect phosphorylation of S6K and AKT, unlike in KINGS-1 cells (Fig. 6C, D). Since mTORC1/2 activities are very high in U87MG, we postulated that the contribution of FilGAP to mTORC1/2 activities may be small. PI3K signaling, one of the upstream activator of mTOR, is known to be activated in glioblastoma by genetic mutations or deletions. So, we examined the effect of FilGAP depletion in U-87MG cells in the presence of PI3K inhibitor (LY294002). PI3K inhibition greatly reduced phosphorylation of AKT and almost completely abolished phosphorylation of S6K. And depletion of FilGAP significantly decreased AKT phosphorylation in U-87MG cells in the presence of PI3K inhibitor (Fig. 6E, F). These results suggest that FilGAP may also be involved in the regulation of mTORC2 in glioblastoma cells.

### FilGAP regulate spheroid growth in KINGS-1 and U-87MG cells

**Figure 7:**
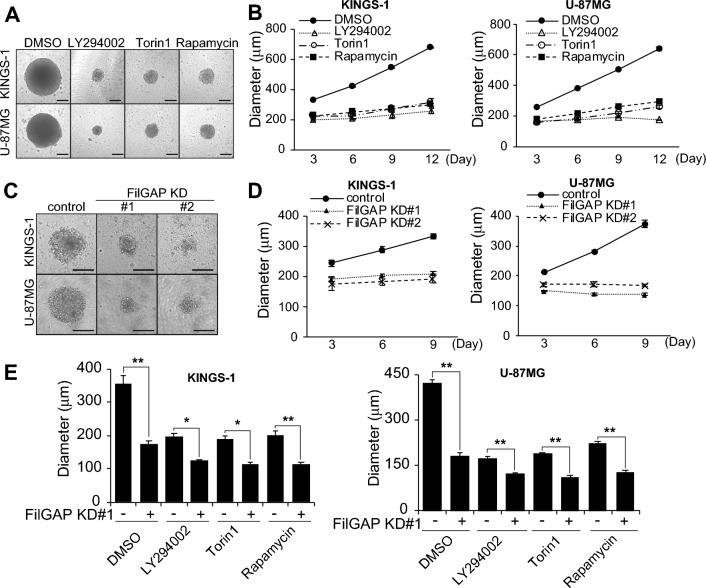
mTOR and FilGAP regulate spheroid growth in KINGS-1 and U-87MG cells. (**A**) Representative images of KINGS-1 and U-87MG spheroids cultured in a round-bottom low attachment 96 well plate, and treated with DMSO, 10 μM LY294002, 500 nM Torin1 or 100 nM Rapamycin for 12 days. Scale bar: 300 μm. (**B**) The diameter of each spheroid of (**A**) was measured (n = 3). (**C**) Representative images of control or FilGAP-depleted KINGS-1 and U-87MG spheroids cultured for 9 days. Scale bar: 200 mm. (**D**) The diameter of each spheroid of (**C**) was measured (KINGS-1; n = 3) (U-87MG; n = 6). (**E**) Control or FilGAP-depleted KINGS-1 and U-87MG spheroids treated with DMSO, 10 μM LY294002, 500 nM Torin1 or 100 nM Rapamycin for 9 days. The diameter of each spheroid on day 9 of was measured (n = 3).

mTOR regulates glioma survival, proliferation, and invasion. To study the role of mTORC1/2 regulation by FilGAP in glioma, we examined whether inhibition of mTOR or depletion of FilGAP affect tumorigenesis using 3D spheroid model of KINGS-1 and U-87MG cells. When KINGS-1 and U-87MG were seeded on low-attachment U-bottom plates, the spheroid size increased with the number of days in culture. We used PI3K inhibitor (LY294002), mTOR inhibitor (Torin1), and mTORC1 inhibitor (Rapamycin) to inhibit mTOR activity. All inhibitors treatment reduced spheroid diameter in both KINGS-1 and U-87MG cells, indicating that mTOR activity is critical for spheroid growth (Fig. 7A,B). Depletion of FilGAP also reduced spheroid diameter in both KINGS-1 and U-87MG (Fig. 7C, D), suggesting that FilGAP promotes spheroid growth in glioma as well as mTOR. Finally, we examined the effects of a combination of mTOR inhibition and suppression of FilGAP expression on spheroid growth. The combination of mTOR inhibitor treatment and suppression of FilGAP expression significantly reduced spheroid size compared to either treatment alone (Fig. 7E, Fig. S3). This suggests that FilGAP may also regulate spheroid growth through pathways other than mTOR pathway. In any case, these data suggest that the combination of mTOR inhibitor treatment and suppression of FilGAP expression has a potent inhibitory effect on tumorigenesis.

## Discussion

In this study, we found that FilGAP interacts with mTORC1/2. FilGAP seems to interact with mTORC1 through Raptor and with mTORC2 through Rictor or Sin1 (Fig. 1 and 2). We also found that FilGAP regulates mTORC1/2 activities in glioma cells. PH domain (1-130aa) of FilGAP, especially the region 96-130aa, is important for mTORC2 activation (Fig. 4, 5). We further show that FilGAP may regulate mTORC2 in the presence of PI3K inhibitors in glioblastomas. Finally, we showed that the suppression of FilGAP expression and mTOR inhibition attenuated spheroids growth in both KINGS-1 and U-87MG. Taken together, these results suggest that FilGAP may regulate tumor growth in gliomas through the regulation of mTORC1/2 activities.

We showed that FilGAP interacts with mTORC1/2 and regulates their activities. However, it is still unclear how FilGAP regulates mTORC1/2 activities. mTORC1 is activated by amino acids and other nutrients though the Rheb and Rag GTPases^4^. On the other hand, mTORC2 is activated primarily by growth factors through PI3K signaling^50^. One possibility is that FilGAP may promote complex formation of mTORC1 and mTORC2. In addition, there is a crosstalk pathway between mTORC1 and mTORC2 that regulates each other's activity^44^. Since FilGAP binds to both mTORC1 and mTORC2, it may be involved in the regulation of mTORC1 and mTORC2 cross-talk. Future clarification of the detailed pathway of mTOR activation by FilGAP may shed light on the very complex regulatory mechanism of mTOR pathway.

We showed that FilGAP transcript isoform partially lacking PH domain, which are highly expressed in gliomas, also activate mTORC1 and mTORC2 (Fig. 5). Knockdown of FilGAP isoform 1 alone increased phosphorylation of AKT and S6K. The increase in phosphorylation of AKT and S6K may be due to the increase of FilGAP isoform 3 or 4. Interestingly, RNAseq data analysis showed that only isoform3 was upregulated in glioblastoma, even though the overall expression of FilGAP decreased with glioma malignancy. This increase of FilGAP isoform 3 may contribute to the malignant progression of gliomas, but this is an issue to be analyzed in the future. Further 96-130aa of FilGAP may be important for mTORC2 activation (Fig. 4, 5). FilGAP 96-748aa corresponding to isoform 3 was suggested to bind Rictor more strongly than ΔPH (131–748 aa) (Fig. 5J). It is possible that FilGAP 1-95aa acts to interfere with the interaction with Rictor. FilGAP with PH domain can also activate mTORC2, it is possible that the binding of PIP3 to the PH domain of FilGAP affects its interaction with mTORC2. Since FilGAP 1-95aa contains Arginine 39, which is important for binding to PIP3, it is considered that FilGAP isoform 3 cannot bind to PIP3^45^. Therefore, it is likely that FilGAP isoform3 partially lacking PH domain can interact with mTORC2 in a PIP3-independent manner.

Depletion of FilGAP in U-87MG, grade IV glioblastoma cell line, did not affect mTORC1/2 activities (Fig. 6). Since highly malignant glioma such as glioblastoma acquires high mTORC1/2 activities by the gene mutations, the contribution of FilGAP to mTORC1/2 activities seems to be reduced. Actually, U-87MG cells have mutations in PTEN, a negative regulator of mTOR pathway^51,52^. However, depletion of FilGAP in U-87MG decreased mTORC2 activity in the presence of PI3K inhibitors (Fig. 6E, F). This suggests that FilGAP may regulate mTORC2 activity PI3K-independent manner. PI3K-independent activation of mTOR has been reported in drug-resistant cancer cells^53^. FilGAP may be involved in the acquisition of drug resistance by cancer cells with high PI3K activity. Remarkably, glioma cells express high levels of FilGAP isoform 3 and 4, which structurally appears to function in a PIP3-independent manner. FilGAP transcript isoform lacking a part of PH domain may be important for PI3K-independent survival of cancer cells. This may be the reason that depletion of FilGAP suppresses spheroid growth not in KINGS-1 cells but also U-87MG cells (Fig. 7C, D). Since the low adhesion and hypoxic conditions during spheroid formation affect the PI3K pathway^54^, the regulation of mTORC1/2 activities by FilGAP might be prominently observed.

This study provides FilGAP as a new mTORC1/2 activator. FilGAP may become a potential therapeutic target for drug-resistant glioblastoma. It will be important issue to be solved to determine how FilGAP activates mTORC1/2, and the role of this regulation in vivo*.*

## Methods

### Cell culture, reagents and antibodies

KINGS-1 cells (Grade III astrocytoma, HSRRB) were maintained in Roswell Park Memorial Institute (RPMI) 1640 supplemented with 10% fetal bovine serum (FBS), 50 units/ml penicillin and 50 mg/ml streptomycin. U-87MG (Glioblastoma, ATCC) cells were maintained in Eagle's minimal essential medium (E-MEM) supplemented with 10% FBS, 50 units/ml penicillin and 50 mg/ml streptomycin. HEK293T cells (ATCC), MDA-MB-231cells (ATCC), and A549 cells (ATCC) were maintained in Dulbecco’s modified Eagle’s medium (DMEM) supplemented with 10% FBS. A7 cells were provided from Dr. Thomas P. Stossel (Harvard University). A7 cells were maintained in Minimum Essential Medium Eagle supplemented with 8% newborn calf serum, 2% fetal calf serum, 50 units/ml penicillin and 50 μg/ml streptomycin. Reagents, antibodies, siRNA used in this study were listed in supplementally table 1.

### Transfection

KINGS-1 cells were transfected with plasmid DNA for 24 h using Lipofectamine 2000 (Invitrogen) according to the manufacturer's instructions. HEK293T cells was transfected with plasmid DNA for 24 h using Poly-Ethylene-Imine (PEI, polyscience). KINGS-1, U-87MG cells were transfected with FilGAP siRNA for 48 or 72 h using Lipofectamine RNAiMax (Invitrogen) according to the manufacturer’s instructions. The cells were maintained at 37 °C with 5% CO_2_ during the treatments.

### Immunoblotting

Total cellular proteins were harvested using RIPA buffer [20 mM Tris‐HCl (pH7.5), 120 mM NaCl, 1% TritonX-100, 0.5% sodium deoxycholate, 0.1% sodium dodecyl sulfate, 10 mM MgCl_2_, 1 mM EDTA, 10 mM NaF, 10 mM β-glycerophosphate, Protease inhibitor Cocktail and 1 mM DTT]. Cell lysates were separated by SDS‐PAGE and transferred to the membrane for fluorescence (Millipore). The membrane was blocked with Intercept (PBS) Blocking Buffer (LI-COR) and incubated with primary antibodies. It was then incubated with secondary antibodies and detected with FUSION SOLO S (Vilber Loumat).

### GST pulldown assay

HEK293T cells were washed with phosphate-buffered saline (PBS), suspended with CHAPS buffer (50 mM Tris–HCl [pH 7.5], 0.1 M NaCl, 2 mM MgCl_2_, 0.1 mM EDTA [pH 8.0], 0.3% CHAPS, Protease inhibitor Cocktail (SIGMA), and 1 mM DTT). The cells were disrupted and the cell lysates were prepared by centrifugation for at 15,000 rpm for 5 min at 4 °C. The supernatant fluid was incubated with GST-FilGAP protein coupled with glutathione-Sepharose beads for 60 min at 4 °C. The beads were washed three times with CHAPS buffer and bound Raptor, Rictor or mTOR were detected by SDS-PAGE followed by Western blot using corresponding antibody.

### Immunoprecipitation

HEK293T cells or KINGS-1 cells were washed with PBS, suspended with CHAPS buffer. Cell lysates were precleared and supernatant fluid was incubated with monoclonal anti-Flag agarose beads or anti-FilGAP bound proten G Sepharose beads for 1 h at 4 °C. Immunoprecipitates were washed three times with CHAPS buffer and bound proteins were detected by SDS-PAGE followed by Western blot.

### Plasmids

The FilGAP siRNA KD#2-resistant construct (rKD#2) was generated by introducing point mutations at nucleotide positions 771, 777, 780, 786, and 792 of the FilGAP coding sequence using the QuikChange site-directed mutagenesis kit (Stratagene, La Jolla, CA). Sin1 was cloned from HEK293T cells by RT-PCR, and subcloned into pCMV5-Flag, pCMV5-Myc and pMAL-c2X. FilGAP, Raptor and Sin1 deletion mutants were generated by PCR. pEF-Bos-Flag-Rictor, pRK5Myc-Raptor was gifted from T Sato (Aichi Cancer Research Institute).

### RT-PCR

cDNA was synthesized from 0.5 μg of total RNA by ReverTraAce (TOYOBO) and amplification was carried out using specific forward primers for *ARHGAP24* gene as follows^46^: isoform 1 primer located: 5′-CTG CAA TGA AGA GAA CCC AG-3′, isoform 2: 5′-ATG CCT GAA GAC CGG AAT TC-3′, isoform 3: 5′-CAG TGG ACA GTT AAA CAA GAG-3′, and isoform 4: 5′-GTCACTGACCACTGAAGTGT-3′. Common reverse primer located 5′-CAT AAC GAA CAG TAT CCT CCA G-3′. β-actin was used as a loading control.

### TCGA RNA sequencing data collection and analysis

Expression data of FilGAP isoforms from the Cancer Genome Atlas (TCGA) was extracted using the TSVdb webtool (http://www.tsvdb.com)^48^. Normalized RSEM (RNA-Seq by Expectation aximization) count estimates from the TCGA Lower Grade Glioma (TCGA-LGG) and Glioblastoma multiforme (TCGA-GBMs) projects were extracted and matched to clinical data on grade downloaded from the TCGA database and sample ID. Expressions level of FilGAP isoforms were compared in data from 67 grade II astrocytomas, 130 grade III anaplastic astrocytomas and 166 GBMs.

### Spheroid culture

For spheroid generation, 200 μl/well of control or FilGAP-depleted KINGS-1 and U-87MG cells suspensions at optimized densities (0.5 × 10^4^ cells/mL) were dispensed into 96-well round-bottomed ultra-low attachment surface plates (Corning or Greiner). Spheroids were incubated for 9 days at 37 °C, 5% CO_2_ with 50% medium exchange every 3 days. Images of the spheroids were acquired every 3 days and their diameters were measured with Image J.

### Supplementary Information

Supplementary Information.

## Data Availability

The datasets generated and analysed, and full sets of results obtained during the current study are available from the corresponding author on reasonable request.
