# Supplementary material for: FilGAP regulates tumor growth in Glioma through the regulation of mTORC1 and mTORC2
Source: Sci Rep. 2023 Dec 8;13:20956. doi: 10.1038/s41598-023-47892-1 (PMC10709582; doi:10.1038/s41598-023-47892-1)
Supplement: Supplementary file 1 — Supplementary Information. [file 41598_2023_47892_MOESM1_ESM.pdf]

Supplementary Figures and table

FilGAP regulates tumor growth in Glioma through the regulation of
mTORC1 and mTORC2

Koji Tsutsumi[1‡*], Ayumi Nohara[1*] Taiki Tanaka[1], Moe Murano[1], Yurina Miyagaki[1], and Yasutaka Ohta[1‡]

‡ Corresponding authors: Koji Tsutsumi and Yasutaka Ohta, School of Science, Kitasato University, 1-15-1 Kitasato, Sagamihara, Minami-ku, Kanagawa 252-0373, Japan, Tel: +81-42-778-9410; E-mail: k.tutumi@kitasato-u.ac.jp, yohta@kitasato-u.ac.jp

Supplementary Figure 1

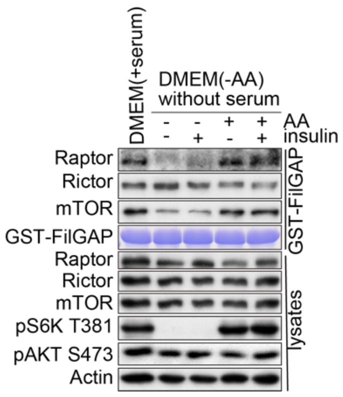

Fig. S1 HEK293T cells were starved in DMEM(-AA) for 2 hours and then incubated with complete medium DMEM (AA) with or without insulin for 30 minutes. Immunoblots for P-S6K1 and P-AKT were presented as the activities of mTORC1 and mTORC2. The purified recombinant GST-FilGAP (373-748aa) or GST alone were coupled to Glutathione-Sepharose 4B, and incubated with HEK293T cells lysed in containing 0.3% CHAPS. The washed precipitates were immunoblotted for the presence of Raptor, Rictor and mTOR.

Supplementary Figure 2

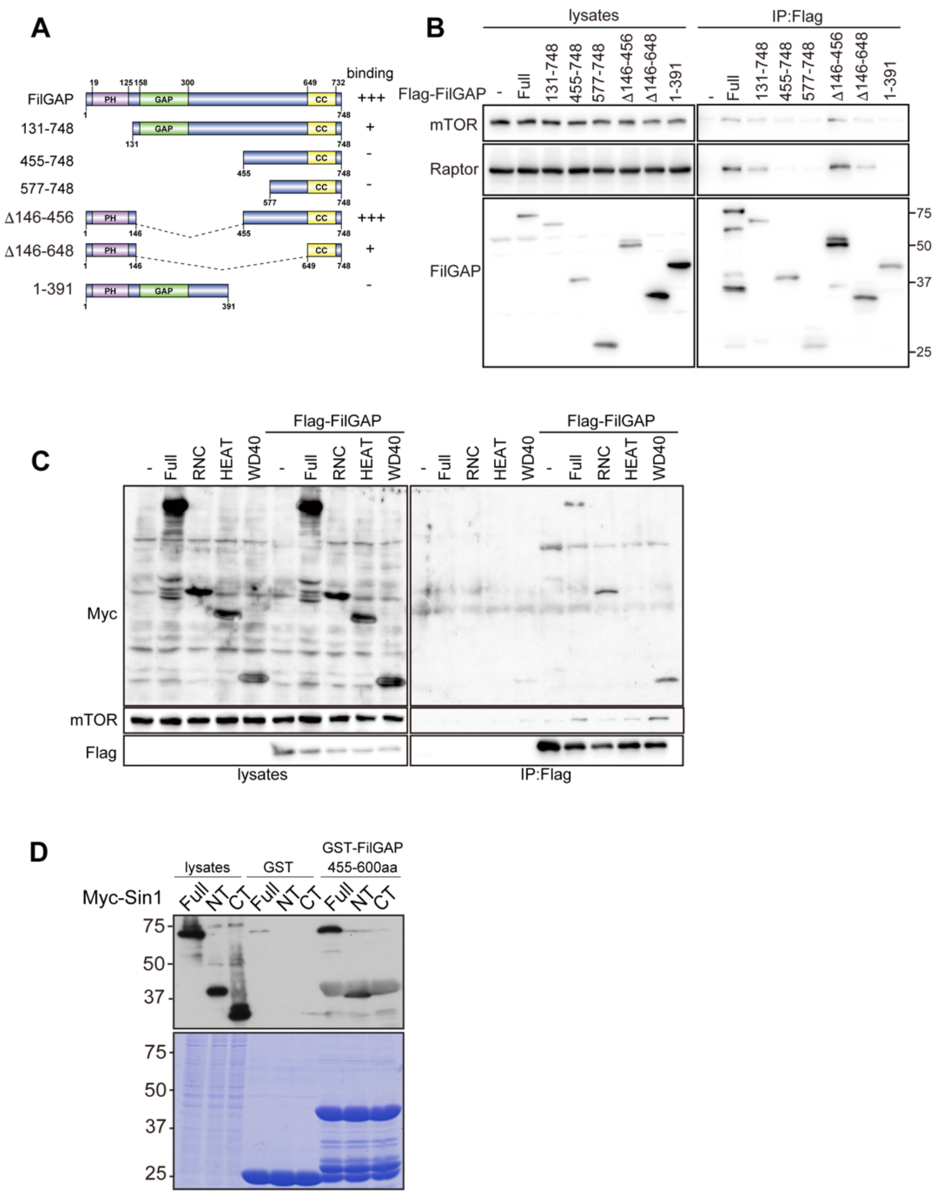

Fig. S2 (A) schematic diagram of Flag tagged FilGAP deletion mutants used to examine the interaction with Raptor by immunoprecipitation. (B) HEK293T cells were transfected with Flag-FilGAP deletion mutants and Myc-Raptor. After precipitation with Flag antibody, coprecipitated Raptor was detected with anti-Raptor antibody. (C) HEK293T cells were transfected with Flag-FilGAP and Myc-Raptor deletion mutants. After precipitation with Flag antibody, coprecipitated Raptor was detected with anti-Raptor antibody. (D) HEK293T cells were transfected with Myc-Sin1 (Full, NT, CT). Cell lysates were prepared from transfected cells and incubated with GST-FilGAP coupled to glutathione sepharose, and the washed precipitates were immunoblotted for the presence of Sin1 using anti-Myc antibody.

Supplementary Figure 3

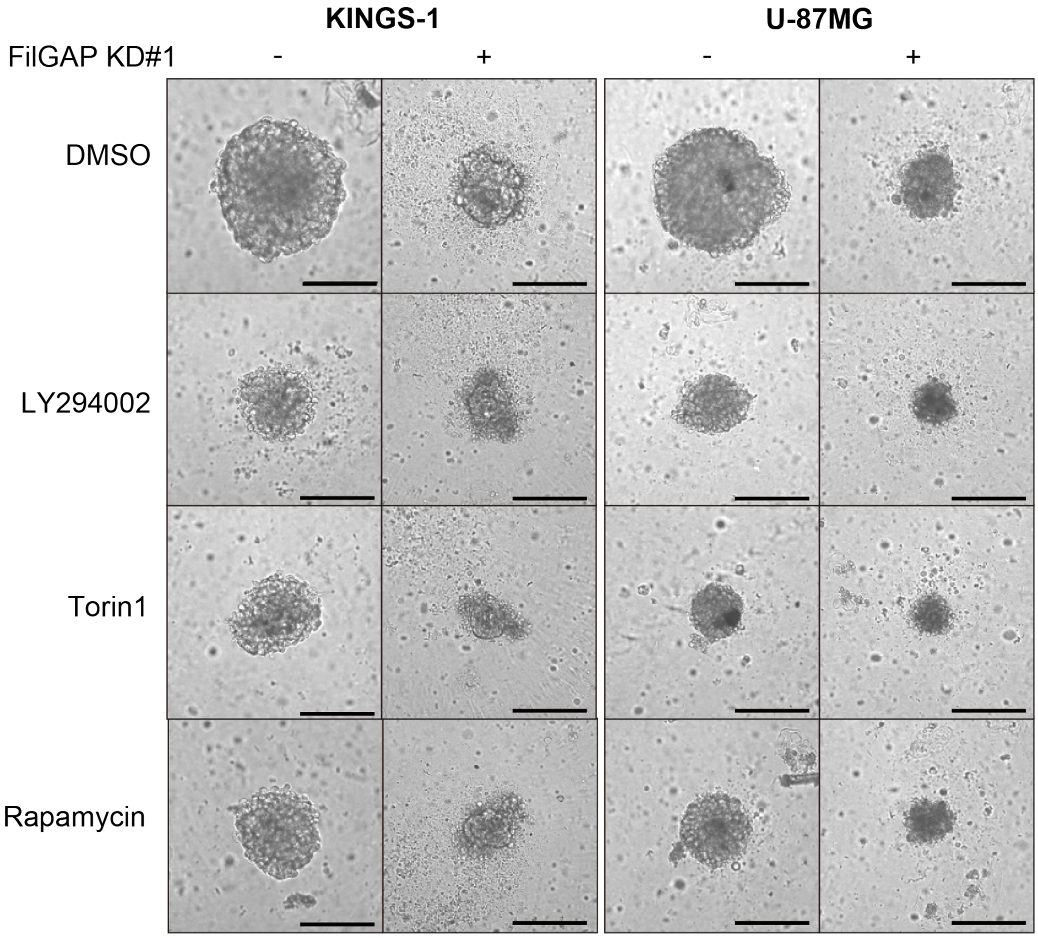

Fig. S3 Control or FilGAP-depleted KINGS-1 and U-87MG spheroids treated with DMSO, 10 μM LY294002, 500 nM Torin1 or 100 nM Rapamycin for 9 days. The images show the representative spheroids on day 6. Scale bar: 200 μm.

Fig. S4 Original uncropped blots

After confirming the position with molecular weight markers, the membrane was cut at the position of the molecular weight of the protein of interest before reacting with the antibody. Areas used in the figures in main text are surrounded by dotted squares.

Supplementary Figure 4

Figure 1

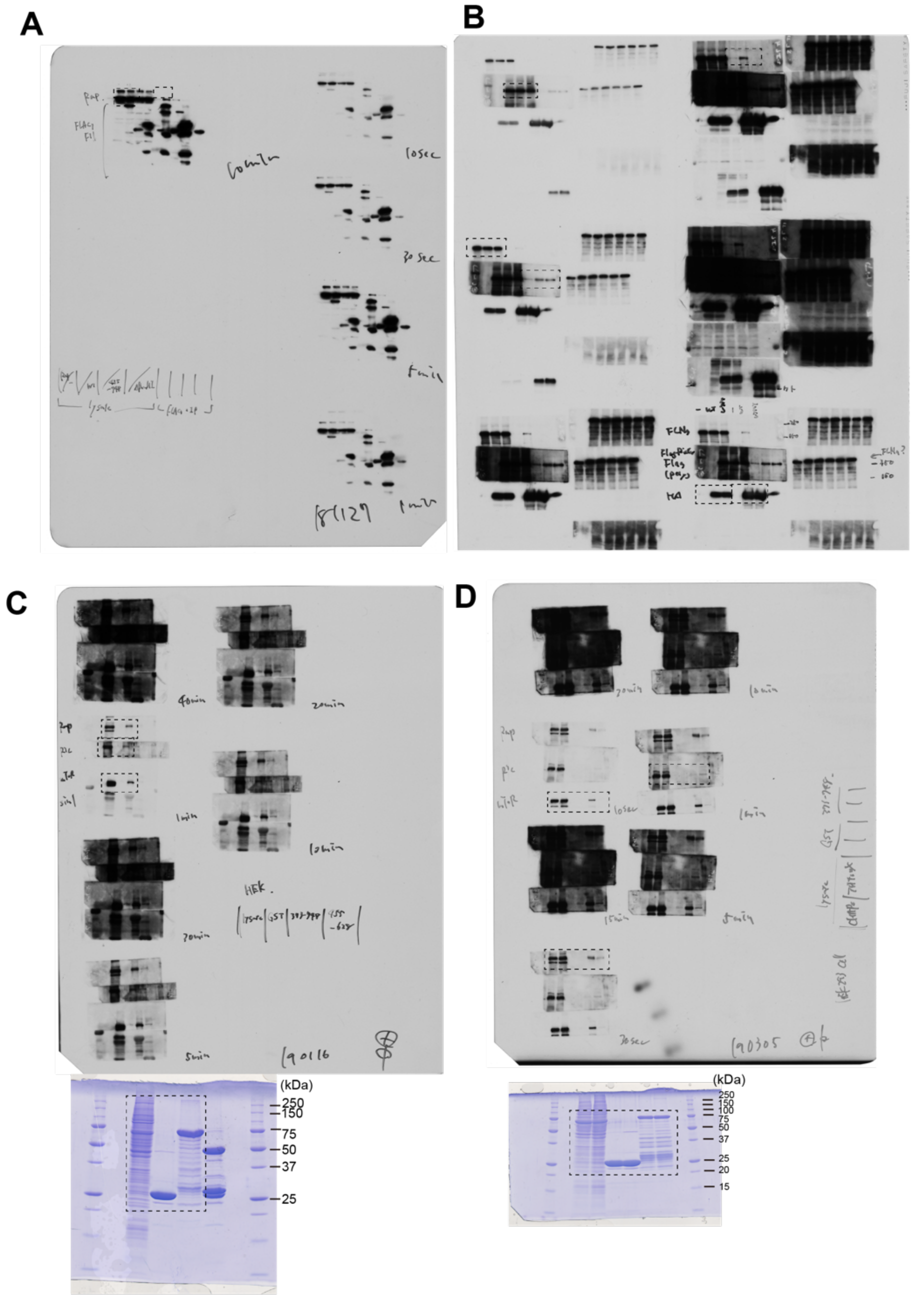

Supplementary Figure 4 continued

F

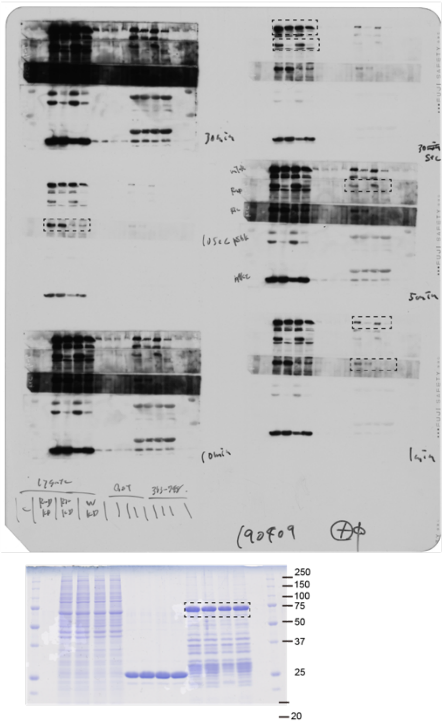

G

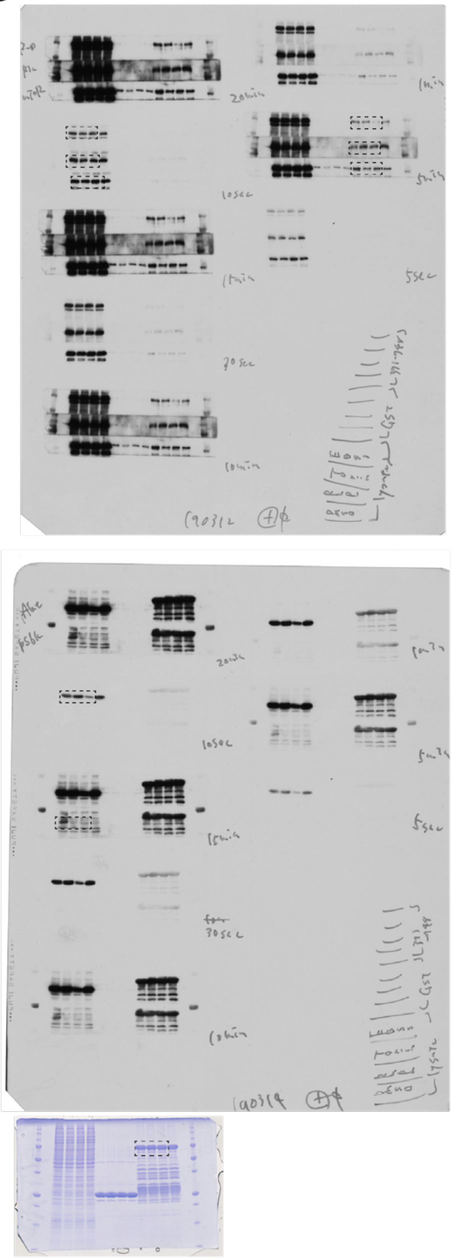

I

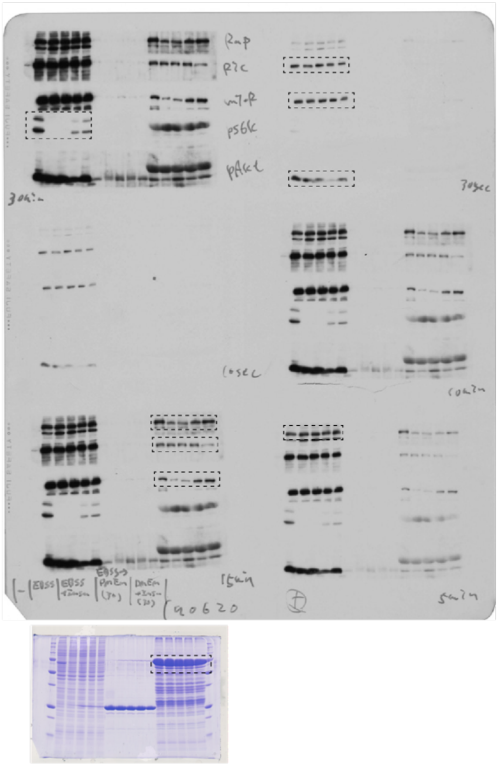

Supplementary Figure 4 continued

Figure 2

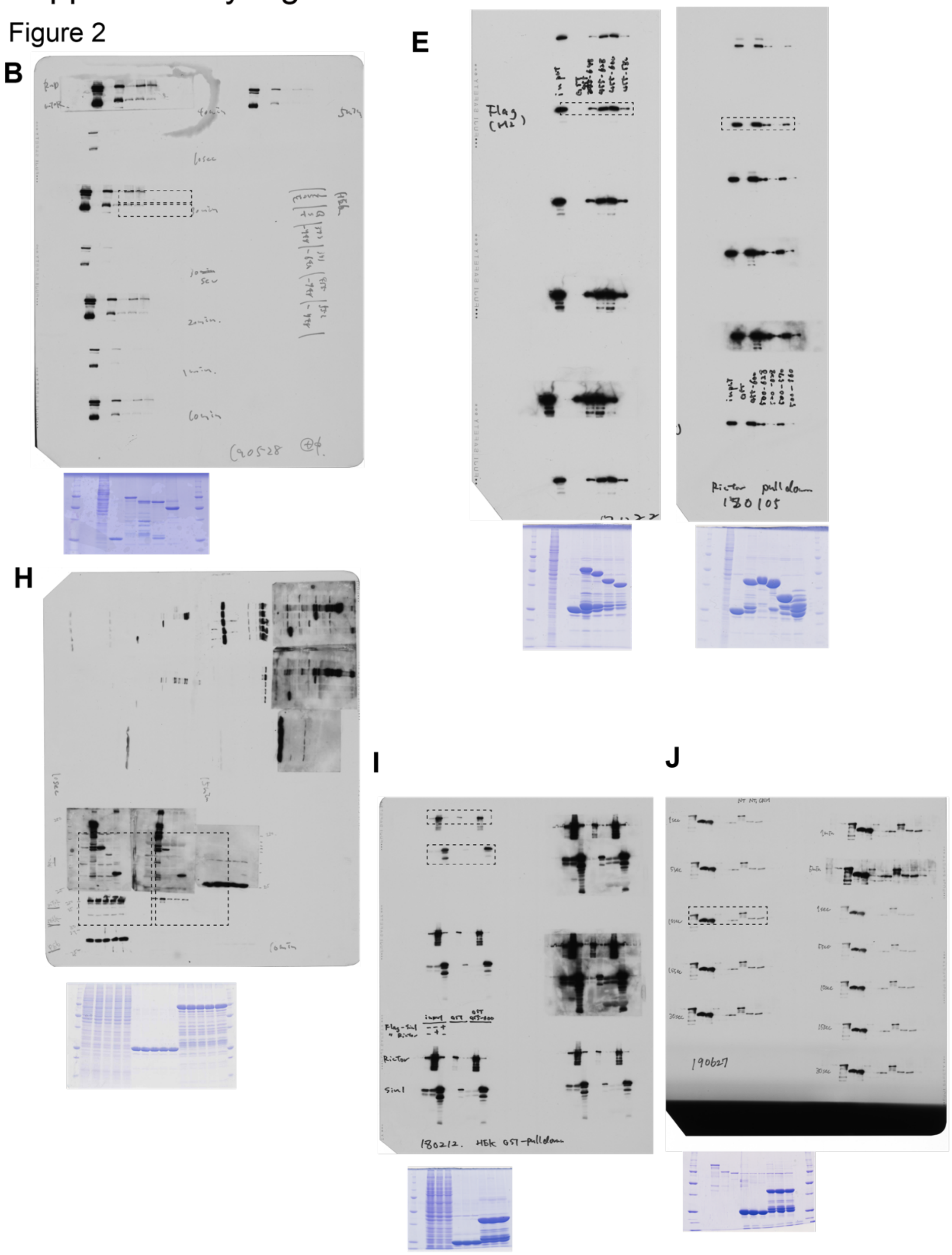

Supplementary Figure 4 continued

Figure 3

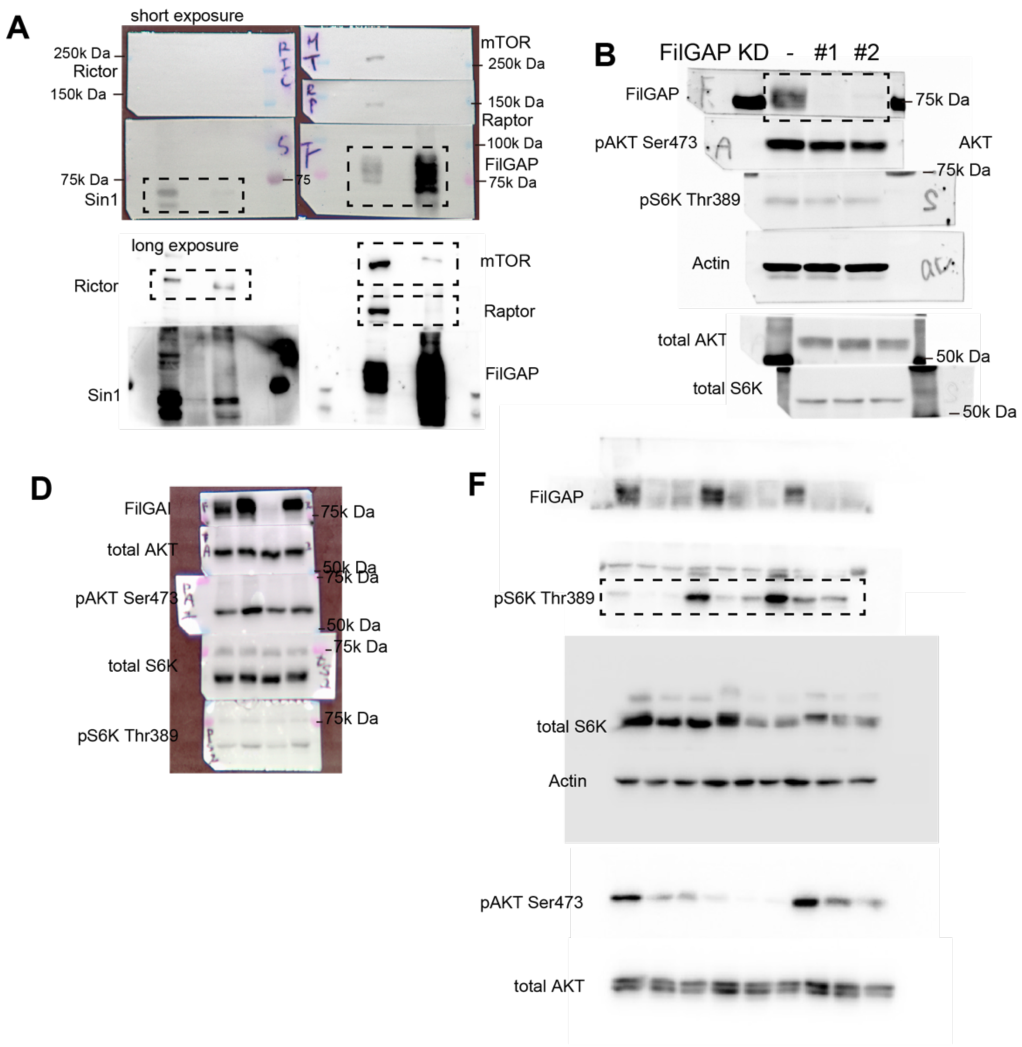

Supplementary Figure 4 continued

Figure 4

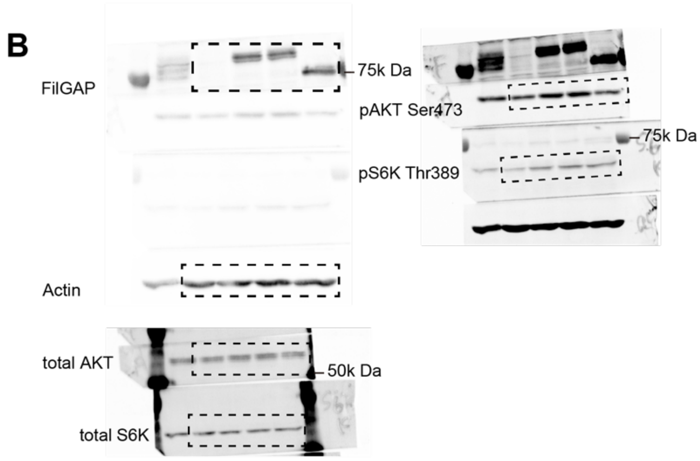

Supplementary Figure 4 continued

Figure 5

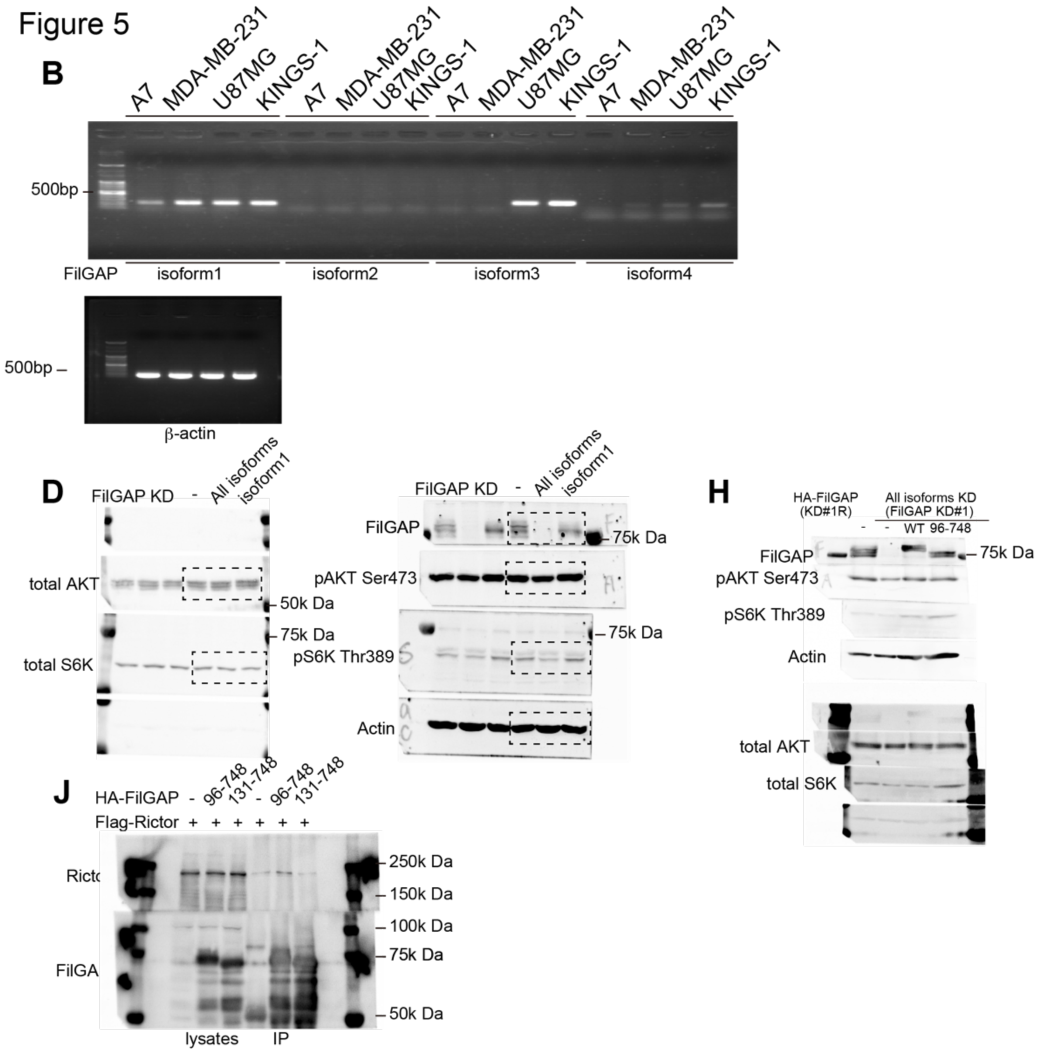

Supplementary Figure 4

Figure 6

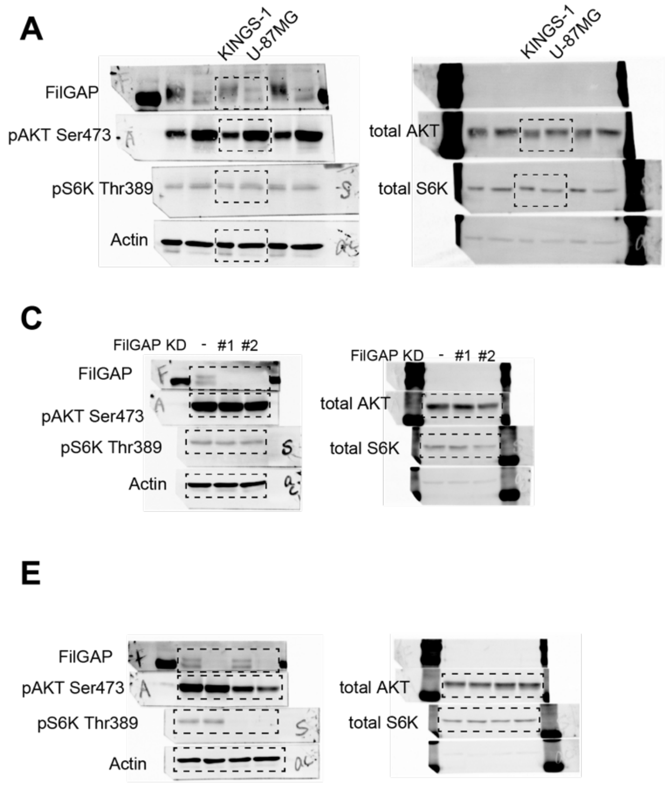

Supplementary Table 1

Name	Company, product number, host	Dilution
Antibody		
anti-DDDDK-tag	MBL, PM020, Rabbit	1:1,000
anti-Flag-tag M2	Sigma, F3165, Mouse	1:1,000
anti-HA-tag	MBL, 561, Rabbit	1:1,000
anti-HA-tag 12CA5	Roche, 11583816001, Mouse	1:1,000
anti-Myc-tag	MBL, 562, Rabbit	1:1,000
anti-phospho-AKT Ser473 D9E	Cell Signaling, 4060, Rabbit	1:2,000
anti-AKT(pan) 40D4	Cell Signaling, 2920, Mouse	1:1,000
Phospho-p70 S6 Kinase (Thr389) 108D2	Cell Signaling, 9234, Rabbit	1:1,000
p70 S6 kinase/S6K1 H-9	Santa cruz, sc-8418, Mouse	1:500
anti-beta-Actin 13E5	Cell Signaling, 4970, Rabbit	1:1,000
anti-alpha-Tubulin B-5-1-2	Sigma, T5168, mouse	1:5,000
anti-mTOR 7C10	Cell Signaling, 2983, Rabbit	1:1000
anti-Raptor	Bethyl labratories, A300-553A, Rabbit	1:1000
anti-Rictor	Bethyl labratories, A300-459A, Rabbit	1:1000
anti-Sin1 1C7.2	Merck, 05-1044, Mouse	1:1000
anti-FLNa PM6/317	Millipore,	1:1000
anti-FilGAP	Ohta et al., 2006, Rabbit	1:500
Goat anti-Mouse IgG(H+L)-HRP	BioRad, 1706516	1:5000
Goat anti-Rabbit IgG(H+L)-HRP	BioRad, 1706515	1:5000
CF800 Goat-anti-mouse IgG	Biotium, 20831, Mouse	1:10000
CF680 Goat-anti-mouse IgG	Biotium, 20065, Mouse	1:20000
CF800 Goat-anti-rabbit IgG	Biotium, 20832, Rabbit	1:10000
CF680 Goat-anti-rabbit IgG	Biotium, 20067, Rabbit	1:20000
Reagent		
Protease inhibitor cocktail	Sigma, P8340	
Rapamycin	LKT labs, R0161	
Torin-1	Sigma, 475991	
LY294002	Sigma, L9908	
Insulin, Human, recombinant	Wako, 093-06471	
KOD-Plus-Neo	TOYOBO, KOD-401	
ReverTra Ace® qPCR RT Master Mix	TOYOBO, FSQ-201	
Penicillin-Streptomycin (5,000 U/mL)	Thermo Fisher, 15070063	
DMEM	Sigma, D6429	
MEM	Sigma, M4655	
RPMI 1640	Gibco, 24010-043	
E-MEM	Fujifilm, 051-07615	
EBSS	Gibco, 22400-105	
Intercept (PBS) Blocking Buffer	LI-COR, 927-70001	
Lipofectamine 2000	Thermo Fisher, 11668019	
Lipofectamine RNAiMAX	Thermo Fisher, 13778150	
Anti-Flag M2 affinity agarose gel	Sigma, A2220	

name	5' to 3' sequence	
siRNA		
human FilGAP KD#1	AAGAUAGAGUAUGAGUCCAGGAUAA	nt 1975-1999
human FilGAP KD#2	CAGUGGUAAAUUACAACCUCCUCAA	nt 789-813
human FilGAP iso1 KD	UCUCCGUGGAGUCAUUGUUCUCCUC	nt 453-477
Raptor KD	UUUCGAAACAGACUAGCCACCAGCA	nt 1022-1046
Rictor KD	UCAUCUUUCUGACUAAUCGAAGUGC	nt 458-482
